# Psychological Autopsy in Small Penis Syndrome: Determining the Manner and Circumstance of Death Following a Vehicular Accident

**DOI:** 10.7759/cureus.42041

**Published:** 2023-07-17

**Authors:** Raveendran Sathasivam, Prasan Wijetunga, BM Munasinghe, B. Dimuthu Mahendra

**Affiliations:** 1 Forensic Medicine, Base Hospital, Panadura, LKA; 2 Anaesthetics, Postgraduate Institute of Medicine, Colombo, LKA; 3 Psychiatry, General Hospital, Vavuniya, LKA

**Keywords:** motor vehicle collision, psychological autopsy, accident reconstruction, small penis anxiety, body dysmorphic disorder

## Abstract

Road traffic accidents and related deaths are on the rise, especially in developing countries. Even though uncommon and probably under-recognized, predisposing psychiatric states may be contributory, posing the question of whether these could be avoided. Psychological autopsy, despite not being performed routinely, might play a pivotal role in such instances, in reconstructing events leading to the catastrophe and determining the role played by the parties involved. The legal implications, such as the exoneration of the wrongly accused, might be substantial. This case report presents a death of a middle-aged South Asian male with strong history and evidence of small penis anxiety following a direct collision in a road traffic accident. It highlights the careful evaluation of the clinical history of the deceased by a psychological autopsy and reiterates the importance of the former in suspected cases.

## Introduction

Motorcycles have emerged as a viable alternative mode of transportation due to convenience, affordability, and economical fuel consumption [1]. Motorcycle-related accidents pose a significant concern, resulting in 806 fatalities and 5,133 cases of disability in 2022 in Sri Lanka, with over two deaths reported daily [2]. Universally, epidemiological research has identified physical illness, alcohol intoxication, and drug use of drivers as contributing factors to road traffic accidents [3,4]. The impact of psychiatric disorders as a risk factor is often unrecognized or under-reported, particularly when riders succumb to traffic-related injuries. Simulating road accident scenarios play a pivotal role in determining and reconstructing the events that led to the fatality and the respective role played by those involved [5]. Recognizing the effect of the driver's physical and psychiatric status on accidents is as important and may determine their liability in legal proceedings [6]. The latter may not be assessed or recognized as contributory during a conventional autopsy following a fatality. In this report, we present a suspected case of body dysmorphic disorder (BDD) known as small penis anxiety (SPA), also known as a penile dysmorphic disorder, and its relevance in determining the manner of death and reconstructing the events following a fatal motorcycle accident in a middle-aged South Asian male. To the best of our knowledge, there have been no reported forensic autopsy cases illustrating the association between SPA and fatal road traffic accidents.

## Case presentation

The medico-legal morgue of a tertiary care hospital in Sri Lanka received the body of a 48-year-old man who had been involved in a motor vehicle collision. According to the history provided, the man died due to injuries sustained in a front-to-front collision with a lorry while riding a motorbike. It was discovered that he was not wearing a helmet or perhaps his helmet was lost during the accident. The lorry driver stated that he was driving the vehicle with control and was not anticipating the collision. It was noted that the motorbike rider could have avoided the accident by turning the motorbike to the side which he failed to do. The other witnesses and the police report back the lorry driver's statement, but it remains uncertain whether it was accidental or suicidal in nature.

Additionally, it was revealed in the history that the deceased had been suffering from an unidentified psychiatric illness (probably BDD) since his third decade of life. He had concerns about the size of his penis, which led him to spend a significant amount of time at home in front of the mirror. Symptoms, such as restlessness, easy fatigue, tiredness, loss of sleep, and muscle tension, were present. He had been having erectile dysfunction since his early adult life, probably related to his preoccupation with his penile size and performance anxiety. However, there was not enough evidence to suggest undue preoccupation with any other body parts. These symptoms worsened intermittently, and he sought treatment from various general practitioners who prescribed unknown medication. He was advised to see a consultant psychiatrist, but he refused due to his belief that the illness was physical rather than psychological, as well as the social stigma associated with seeking help.

There were no known physical illnesses in his medical history, such as hypertension, uncontrolled diabetes, ischemic heart disease, epilepsy, or other neurological disorders. He had not undergone any surgeries, but he frequently visited places in hopes of finding remedies for his concerns about the size of his penis. These details were unknown to the witnesses.

The history also revealed features of depression and suicidal ideation, although there were no suicide attempts. His social life was disrupted, and he was dismissed from the police force due to frequent absences from work. He was married and had two children but was estranged from his family for an extended period. There was no evidence to suggest any paraphilia. He had been having features of alcohol dependence syndrome. There was no history of other sedative or addictive drug use. The government scientist's report on the motorbike and lorry did not indicate any mechanical faults.

Autopsy findings

**Figure 1:**
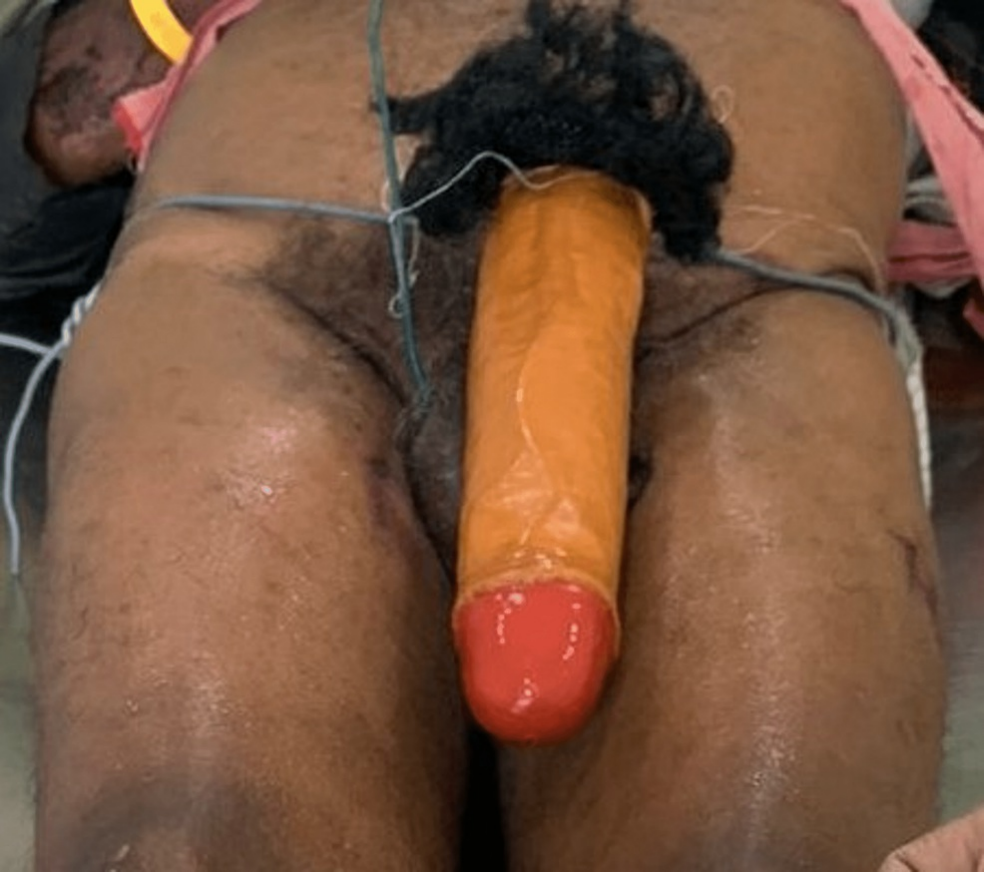
Artificial penis made of fiber material, a wire connecting the artificial penis to the waist

**Figure 2:**
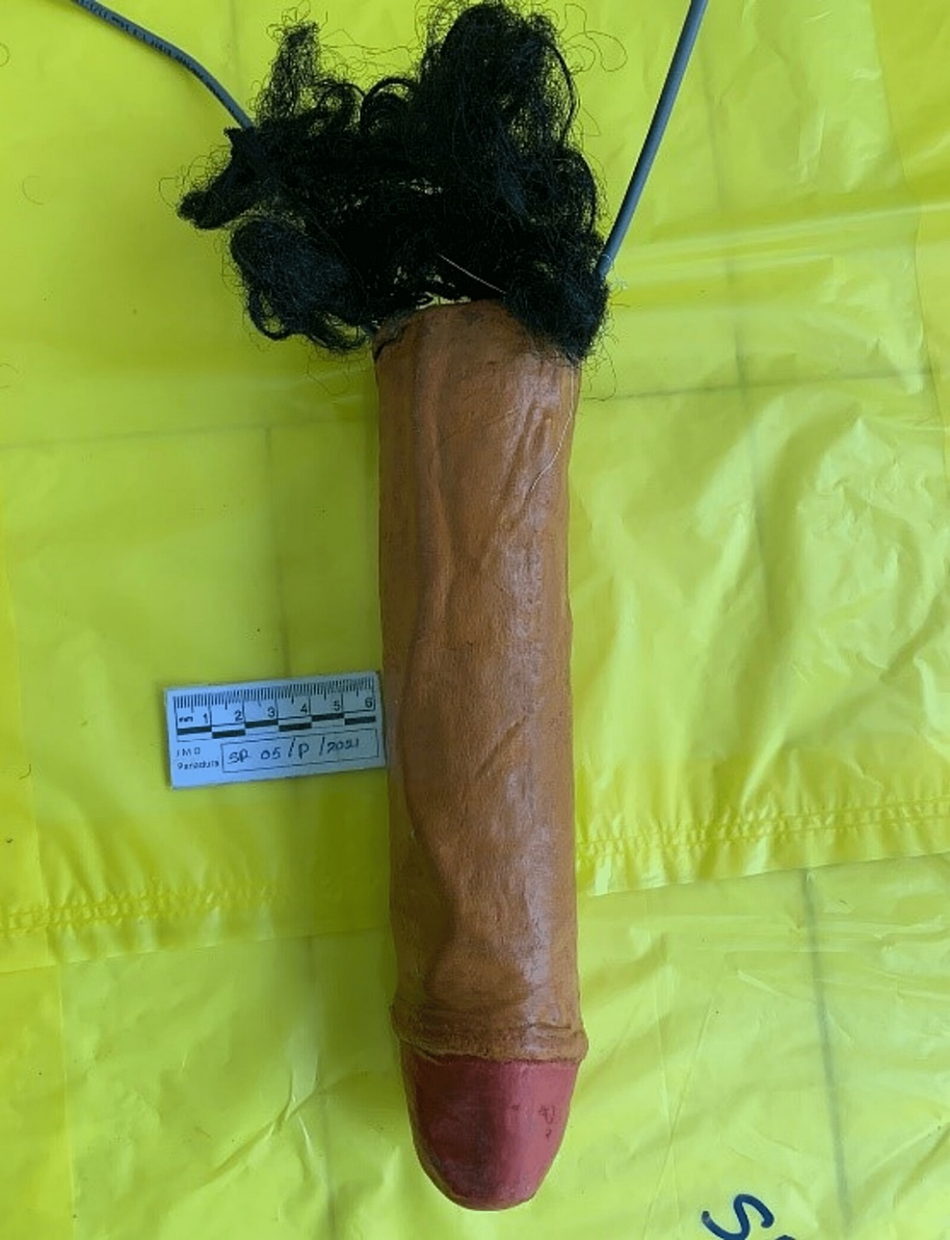
Artificial penis measures length of 26 cm and diameter of 5.5 cm, a bunch of artificial hair

The body mass index of the victim was 24. External examination revealed injuries which included scalp laceration, impact abrasions, and grazed abrasions on the face, body, and upper and lower limbs. The post-mortem interval was 18 hours. Notably, an artificial penis made of fiber, measuring 26 cm in length and 5.5 cm in diameter, was found placed over the anatomical penis, connected to the waist with a wire around the hip. Additionally, a bunch of artificial hair was present (Figures 1, 2).

**Figure 3:**
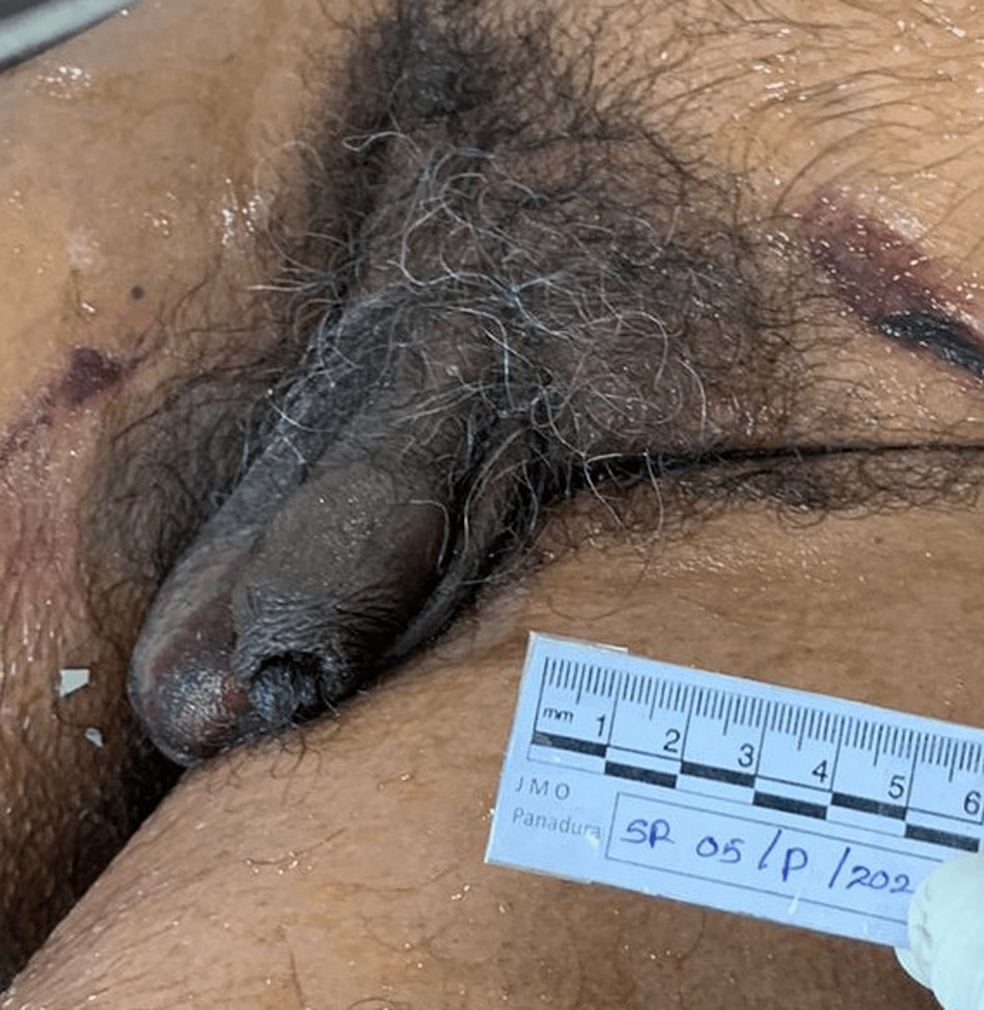
Anatomical penis: the natural penis of the deceased in the flaccid stage, 9.5 cm in length and 4 cm in diameter

The size of the anatomical penis in the flaccid stage was measured at 9.5 cm in length and 4 cm in diameter (Figure 3).

**Figure 4:**
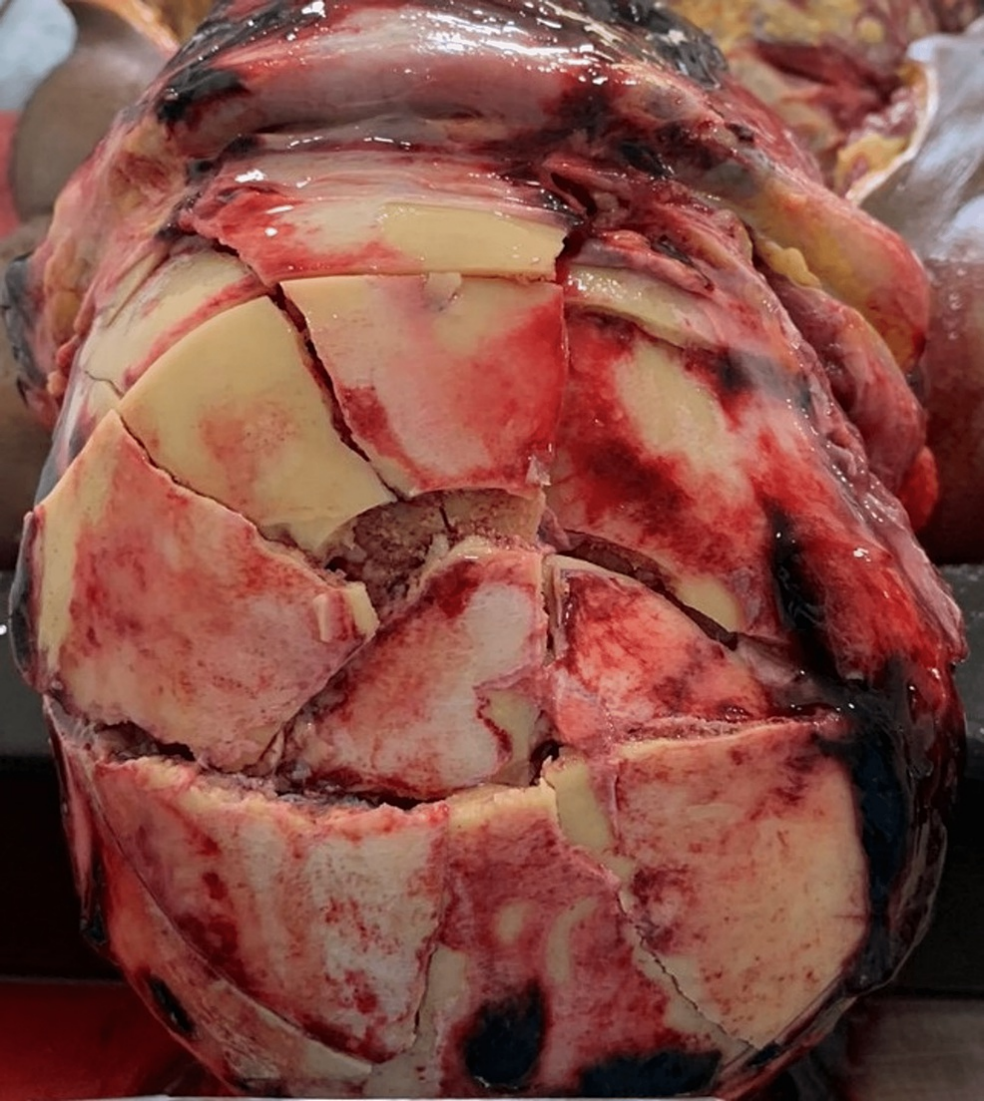
Extensive fractures in the bones of the skull, accumulation of blood beneath the scalp

Internal examination revealed an extensive subscalpal hematoma, multiple skull fractures, a small subdural hemorrhage, and a small laceration on the right frontal lobe (Figure 4).

Further examination of all organs showed no remarkable findings, except for mild calcification and atheroma causing less than 40% luminal narrowing in the left anterior descending artery. There was no evidence of new, healing, or old myocardial infarction (fibrosis), as confirmed by histology.

Toxicological analysis of the blood and urine did not detect alcohol, sedative drugs, or substances of abuse such as heroin, morphine, or cannabis. The cause of death, determined at the conclusion of the medico-legal investigation, was attributed to craniocerebral (head) injuries resulting from blunt force trauma sustained in the motor vehicle collision. Despite the lorry driver's assertion that he was not responsible for the accident, he was detained for further inquiry on the basis of negligent driving.

## Discussion

SPA, also known as "small penis syndrome," a type of BDD, is characterized by anxiety related to one's genitals, either directly or indirectly (while clothed), stemming from concerns about the size of the flaccid penis, despite clinical evidence suggesting otherwise [7]. Men who are distressed about the size of their penis often seek consultations with professionals in urology, andrology, surgery, dermatology, psychiatry, and sexual medicine [8,9]. The syndrome can lead to feelings of inadequacy causing significant distress, anxiety, and depression along with negative work-related and social implications [8].

BDD is defined by a distressing and debilitating fixation/preoccupation with perceived or minor flaws in one's physical appearance. This preoccupation is often accompanied by repetitive and time-consuming behavior [9]. The preoccupation in BDD is most commonly on the face [10]. Occasionally in men, it is focused on their penis size even though their size is within the normal range and no detectable organic causes for a small penis. Penile size is thought to be an important feature of man's nature, sexual competence, and the power of procreation while sexualizing with women. SPA has been described in the literature in men who are dissatisfied or excessively worried about their penis size which is in the normal range [7]. They must also experience clinically significant distress, depression, psychological illness, or impairment in social, occupational, or other important areas of functioning [11]. Excess alcohol intake, sedative drugs, and addictive drugs were not found in the blood, excluding the possibility as a basis for the accident in this patient [3]. Clinical history, circumstantial evidence, and meticulous autopsy examination excluded the possibility of natural illnesses such as hypertension, uncontrolled diabetes, ischemic heart disease, epilepsy, and other neurological diseases as contributory factors [4]. Available clinical history regarding the behavior of the deceased, circumstantial evidence, and findings of an artificially made penis covering the anatomical penis suggest the possibility of psychiatric illness in the deceased, most probably SPA disorder. He had preoccupations about the size of his penis since the early adult period and suffered from psychological consequences indicated by treatment by general practitioners in the past. As he has not been seen by a consultant psychiatrist, no formal diagnosis had been made.

Monograms evaluating flaccid pendulous length with a mean of 9.16 cm and a standard deviation of 1.57 cm and erect length with a mean of 13.12 cm and a standard deviation of 1.66 cm are considered relatively normal for adult penis [12]. In our patient, the penis size was normal, and there was no anatomical or congenital deformity. His penis had been functionally normal as indicated by a previous marriage with two children. However, there is a dilemma regarding whether his two children are actually his own. The fact that his wife separated from him some years ago might be due to his sexual dysfunction. These facts cannot be confirmed due to ethical reasons. His symptoms of loss of sleep, excessive worry, irritability, agitation, and unable to sit in the same place augmented recently indicated that he had been suffering from severe anxiety following persistent worrying thoughts of his small penis. Severe anxiety may affect cognition leading to reduced attention, concentration, and focus and impaired coordination affecting driving skills [13] predisposing to road traffic injuries. It is consistent with the allegation of the lorry driver and circumstantial evidence that the deceased did not take any steps to avoid the collision, even though there was a possibility to prevent the accident by the deceased. SPA can lead to profound anxiety, depression, and a sense of powerlessness that may escalate to suicidal ideation [14]. Such is the case that the possibility of suicide cannot be ruled out completely in this fatal vehicular accident. According to a study by Schmidt et al., it was concluded that 1.7% of all fatal crashes were categorized as suicides, while 1% of nonfatal crashes were identified as attempted suicides [15].

Medico-legal examination of the lorry driver did not reveal any significant illness, and toxicological screenings were negative for alcohol, sedative drugs, and abusive drugs which impair driving skills. According to the government vehicle analysis expert, vehicle examination including lorry and motorbike did not reveal any faults.

The driver of the lorry was remanded to initiate an inquiry according to the current law of Sri Lanka, even though his repeated denial of responsibility for this accident [16]. However, it is important to note that legal systems can vary by country and region, and specific laws or regulations in Sri Lanka may govern the process of remanding drivers involved in accidents without a formal inquiry. In general, the decision to remand a person without inquiry may depend on various factors, such as the severity of the accident, the potential flight risk of the driver, concerns about tampering with evidence, or the need to ensure the safety and security of the public. These decisions are usually made by magistrates or judicial authorities based on the information presented to them [17].

The majority of accident investigation systems globally do not mandate routine psychological autopsy, even though its crucial role in deciding the manner of death and reconstruction of events, as highlighted in previous studies, cannot be undermined [18,19]. Conversely, psychological autopsies are frequently performed in countries such as Australia in suicidal cases [20]. A major limitation of psychological autopsy, in this case, was the uncertainty of the reliability and validity of the information. The absence of a standard methodology, the presence of recall bias, distorted versions from various individuals, and limited collateral records are limiting the accurate diagnosis of any psychiatric illness and its admissibility in the court for the reconstruction of the events. It is insufficient reasoning to correlate a direct connection between sexual dysfunction and a predisposition toward vehicular accidents in a criminal trial where facts should be proven beyond a reasonable doubt. However, the psychological trauma associated with SPA may significantly influence the manner and circumstances of death as discussed earlier. Exploring this aspect can only be accomplished by conducting a psychological autopsy in addition to a pathological autopsy.

## Conclusions

Despite the limited validation of retrospective data collected from the next of kin, medical records, and witnesses, findings of the psychological autopsy could be considered in court if provided with optimal validation. Caution should be exercised when incorporating the findings of a psychological autopsy into the proper reconstruction of events, as these findings may not be as concrete as those obtained from a pathological autopsy. Further research should incorporate psychological autopsy to investigate the role of psychiatric illnesses in death investigations, especially when concerns about pre-existing behaviors influencing the manner and circumstances of death arise, as relying solely on pathological autopsy may be insufficient.
